# Supporting clinicians post exposure to potentially traumatic events: Emergency department peer support program evaluation

**DOI:** 10.1111/1742-6723.14518

**Published:** 2024-10-13

**Authors:** Belinda Carne, Jeremy Furyk

**Affiliations:** ^1^ University Hospital Geelong, Barwon Health Geelong Victoria Australia; ^2^ School of Medicine, Deakin University Geelong Victoria Australia

**Keywords:** critical incident, healthcare personnel, peer support, psychological first aid, secondary traumatic stress

## Abstract

**Objective:**

Workers in EDs are regularly exposed to potentially traumatic events. Since the COVID‐19 pandemic, there has been exponential interest in peer support programs (PSPs) in a range of settings. We describe a PSP implemented in 2017 at University Hospital Geelong (UHG) ED together with results of a survey.

**Methods:**

To describe the program such that others can replicate it in their settings in addition to feedback evaluation. Method involved a survey emailed to the ED doctors.

**Results:**

Thirty responses from 96 emails with a range of feedback.

**Conclusion:**

ED Doctors place high value on the PSP.

## Introduction

The ED Doctors Peer Support Program (PSP) began in 2017 after a critical incident in which investigation preceded support for the healthcare workers involved.^1^

## Description of program

Each craft group in ED has a team of volunteer peer support providers plus team leader who undergo a half day experiential training workshop and can be contacted verbally, by text and by generic email address.

These groups run autonomous programs – doctors for doctors, nurses for nurses and PSAs (patient services attendants), and administration staff for administration staff.

Team members provide support in relation to notified incidents and, in the case of the doctors program, are an adjunct to existing mentor and trainee support structures.

Notifications can be made by any staff member after becoming aware of a potentially traumatic event which includes critical incidents, for example, paediatric death/code blacks in addition to other events with potential for psychological trauma. Notifications received are posted to the team WhatsApp group where a team member then self nominates to contact involved staff members. A generic text message is sent with an opt out option and phone contact usually ensues within 24 h of text. The conversations last between 5 and 30 min on average and are limited to two conversations per event with a post call text being sent with resources for further support as needed.

Team meetings are held on an *ad hoc* basis and the WhatsApp group provides a forum to raise issues between meetings. Conversations are confidential unless mandatory disclosure applies and the components include active listening, empathy and non‐judgement.

## Objective

To evaluate the usefulness of the UHG ED Doctors PSP.

## Methods

Survey conducted *via* Survey Monkey sent to all doctors working in our ED in early 2023. Consisted of 10 questions in relation to demographics and program evaluation with space for optional free text comments and feedback. Survey questions are included in Supporting Information.

## Results

The program was notified of 82 events over 6 years, with over 200 clinician contacts made (some doctors involved in multiple events and a range of 1–23 doctors contacted per event).

The survey was sent to 96 doctors with 30 respondents (31%), consisting of 16 consultants (53%), 12 registrars (40%) and 2 junior doctors (6%). 21 (70%) females. Survey results and selected indicative free text comments are presented in Tables 1 and 2, respectively.

**TABLE 1:** Summary of survey results

Demographics (*N* = 30)	*n*	%	95% CI
Clinician level			
Consultant	16	53	35–71
Registrar	12	40	25–58
Junior doctor	2	7	0–21
Gender			
Female	21	70	52–83
Age			
20–29	3	10	
30–39	14	47	
40–49	11	37	
50–59	2	7	
Previous contact by PSP			
Yes	23	77	59–88

Survey questions	*n*	%	95% CI
Is PSP useful?			
Yes	30	100	89–100
No	0	0	0–11
Optimal time for contact			
<24 h	8	27	14–44
<48 h	19	63	46–78
<1 week	3	10	4–26
Previous experience with PSP			
Positive	22	73	56–86
Negative	0	0	0–11
Neither positive or negative	2	7	2–21
Not applicable	6	20	10–37
Prior critical incident and not contacted by PSP?			
Yes	7/29	24	12–42
No	22/29	76	58–88
Should PSP expand to other stressful work events			
Yes	21	70	52–83
No	9	30	17–48

**TABLE 2:** Selected, indicative comments from respondents

Comment	Staff level
‘*invaluable opportunity to chat about difficult cases after the fact*’	Consultant (F)
‘*I was amazed at how useful I found the discussion, in terms of the emotional support and offloading, plus thinking through clinical aspects*’	Consultant (F)
‘*the sense that there is a team at work in the ED and that others care and have ones back is always a positive and useful thing*’	Consultant (M)
‘*simply knowing that someone would make contact had a great impact on me. Just that knowledge seemed to relieve the stress of what I had witnessed…It meant I didn't feel like I had to deal with it alone if I found myself struggling post the event*’	Junior doctor (F)
‘*Wish there had been this program during my training*’	Consultant (M)
‘*Felt very supported at the time of the incident, and secure in the knowledge of supports*’	Junior doctor (F)
‘*This is so beneficial. I have utilised it once, and then turned it down again when offered. The way this is set out is incredible (if you don't respond I'll call you anyway). Coming from a place where I was involved in many critical incidents and had no support it is wonderful to see*’	Registrar (F)

## Discussion

Our survey shows that the PSP is highly valued by doctors in our department.

This is in line with PSPs in other high trauma areas, for example, police, fire and ambulance services. Interest in their utility is growing exponentially in the medical field.^2^, ^3^

Ours is one of the first PSP programs for ED doctors in Australia and has evolved over time, based on user feedback over 7 years. At program commencement, there was minimal literature in relation to guidelines for PSPs.^4^

In the past, some critical incident support processes had been shown to cause harm in some settings despite an assumption of benefit and there is little existing data describing the efficacy of programs in relation to the mitigation of post critical incident stress.^5^

Our results clearly support the value of our program to doctors.

Limitations include the relatively small number of survey results, a skew towards responses from more senior clinicians, and responder bias.

Reasons for the skew may include the short time junior clinicians spend in the department and a reduced awareness by notifiers of their involvement in critical incidents and potentially stressful events. Addressing this includes education about notifying events and more junior staff being involved as PSP representatives.

Areas of development include expanding the program to other disciplines in the hospital and we are currently providing training for clinicians and allied staff in ICU and anaesthetics where programs have recently commenced.

Further evaluation in relation to PSP training and PSP provider experience are areas for future research in addition to evaluation of the evolving nursing and support staff programs.

## Conclusion

The ED PSP at UHG has been shown to have benefits for doctors in the ED including a sense of being supported, a sense of connection and the opportunity to discuss difficult incidents and their sequelae with a peer who understands their situation.

Our program could be replicated in other similar health care settings across a range of healthcare providers.

## Supporting information


**Data S1:** Supporting Information.

## Data Availability

The data that support the findings of this study are available from the corresponding author upon reasonable request.
