# Supplementary material for: Supporting clinicians post exposure to potentially traumatic events: Emergency department peer support program evaluation
Source: Emerg Med Australas. 2024 Oct 13;37(1):e14518. doi: 10.1111/1742-6723.14518 (PMC11744404; doi:10.1111/1742-6723.14518)
Supplement: Supplementary file 1 — Data S1: Supporting Information. [file EMM-37-0-s001.docx]

**SUPPLEMENTS**

**Text pre call**

Dear (name)

A notification has been received by our ED peer support group that you have been exposed to a potentially stressful event at work.

If you would prefer not to have contact (confidential call) from a designated peer support colleague in relation to this, please text back 'no thanks’ to this phone number.

Otherwise I will aim to make contact in the next day or so.

Kind Regards,

(name)

Website link:

<https://www.psychology.org.au/for-the-public/Psychology-topics/Trauma>
Employee Assistance Program (confidential and free)
1300 687 327

**Text post call**

This is an automated text from the UHG Peer Support Program sent to all participants post call.

There is an option to read the information below and to access care through the Employee Assistance Program (EAP), your GP, Staffcare, psychologist or phone services if wanted.

You are also welcome to arrange a 2^nd^ peer chat by texting this number or by emailing peersupportdocsed@barwonhealth.org.au

Thanks for your ongoing involvement in the care of yourself and others

<https://psychology.org.au/for-the-public/psychology-topics/trauma>
Employee Assistance Program: 1300687327
StaffCare: 42153220

Lifeline: 131114
Beyond Blue: 1300224636

**Peer Support Program Survey Questions**

**Q1:** What is your position in the Emergency Department?

Options: Consultant, Registrar, Resident, Intern, Other doctor
**Q2:** What is your gender?

Options: Male, Female, Other
**Q3:** What age group do you belong to?

Options: 20-29, 30-39, 40-49, 50-59, 60+

**Q4**: Do you think it's useful to have a peer support program that contacts doctors post critical incident?

Options: Yes, No

Optional Comment:
**Q5:** Currently the PSP aims to contact doctors within 48hrs post incident where possible. What time frame do you think is optimal for contact to occur post incident?

Options: Immediately post incident, Within 24 hours, Within 48 hours, Within a week, N/A
**Q6:** Have you been contacted by a PSP colleague after a critical incident?
Options: Yes, No

**Q7:** How did you feel about this contact?
Options: Overall positive, Overall negative, Neither positive nor negative, N/A

Optional Comment:

**Q8:** Have you been involved in a critical incident and not been contacted by a PSP?
Options: Yes, No

Optional Comment:

**Q9:** Would you like to see the program expand to include contact for other potentially stressful workplace events in addition to post critical incident contact?
Options: Yes, No

Optional Comment:

**Q10**: Do you have any feedback in relation to the Peer Support Program?

Optional Comment:
